# Splenic rupture, secondary to G-CSF use for chemotherapy induced neutropenia: a case report and review of literature

**DOI:** 10.1186/1757-1626-1-418

**Published:** 2008-12-24

**Authors:** Nehal Masood, Asim Jamal Shaikh, Wasim Ahmed Memon, Romana Idress

**Affiliations:** 1 Section of Oncology, Department of Medicine, the Aga Khan University Hospital, Karachi, Pakistan; 2 Department of Radiology, the Aga Khan University Hospital, Karachi, Pakistan; 3 Department of Pathology, the Aga Khan University Hospital, Karachi, Pakistan

## Abstract

**Introduction:**

Chemotherapy Induced neutropenia is a frequent and serious complication of cytotoxic cancer treatment.

Granulocyte colony stimulating factors (G-CSF) are frequently used to counter neutropenia, attempt rapid recovery of patients and allow for continuation of treatment without compromise on dose, especially in curative malignancies. Generally regarded as safe, G-CSF use has been very rarely reported to have resulted in serious side effects, such as, splenic rupture.

**Case presentation:**

We are reporting a case of a twenty years old man, who was being treated for T cell acute lymphoblastic leukemia and received colony stimulating factors for treatment of severe neutropenia and suffered from splenic rupture, He was treated with splenectomy.

**Conclusion:**

Although extremely rare, splenic rupture can be a serious and sometimes life threatening complication of high dose colony stimulating factors therapy.

## Introduction

One of the most serious toxicity of chemotherapy is neutropenia, a complication that leads to infection, hospitalization, and even death. Dose reduction in subsequent cycles is also one of the compromise which has to be made in anticipation of troublesome neutropenia[1]. G-CSF therapy promote white blood cell (WBC) proliferation, maturation and functional capacity [2], they are widely used to treat myelosupression caused by chemotherapy. By their effect of reducing the duration and severity of neutropenia, G-CSF use allows for continuation of cytotoxic chemotherapy, that is, in order to obtain superior long term results in various cancers[3]. The results of using G-CSF in primary prophylaxis for neutropenia are promising[4], however their effectiveness in treatment of established neutropenia remains controversial[5]. G-CSF use is the cornerstone of therapy, for hematopoietic stem cell mobilization for stem cell transplantation[3]. Therapy with G-CSF is generally regarded as safe, as side effects with doses as high as 600 μg/day, in healthy volunteers have been tolerated safely. Therapy induced high WBC counts tend to abate within 48 hours of withdrawal [6]. Side effects from long-term use as in patients with congenital neutropenia have also been regarded as generally safe with need of stopping therapy arising rarely if ever[7]. Myocardial infarction, stroke and splenic rupture are some of the rare side effects of high dose G-CSF therapy. There are few reported cases of splenic rupture secondary to use of G-CSF so far, most of the reported patients were either healthy donors of stem cell transplant patients or patients undergoing peripheral blood stem cell mobilization for transplant (PBSCT) [2].

We here in report a case of a young patient suffering from T-cell lymphoblastic lymphoma, who received G-CSF as secondary prophylaxis and unfortunately suffered from splenic rupture.

## Case report

**Figure 1:**
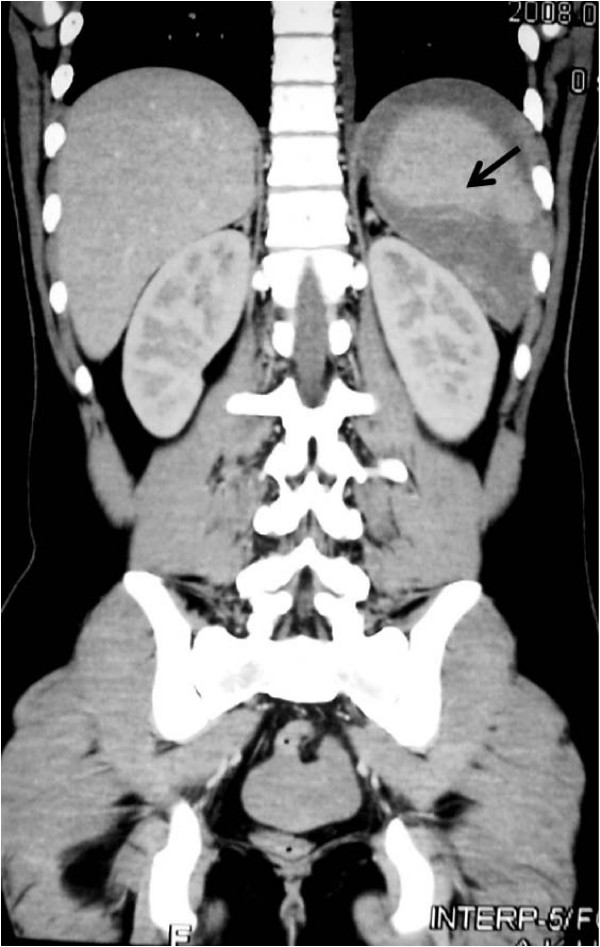
**CT SCAN Axial cuts showing peri-splenic fluid collection (bleed) and Niche of rupture (arrow)**.

**Figure 2:**
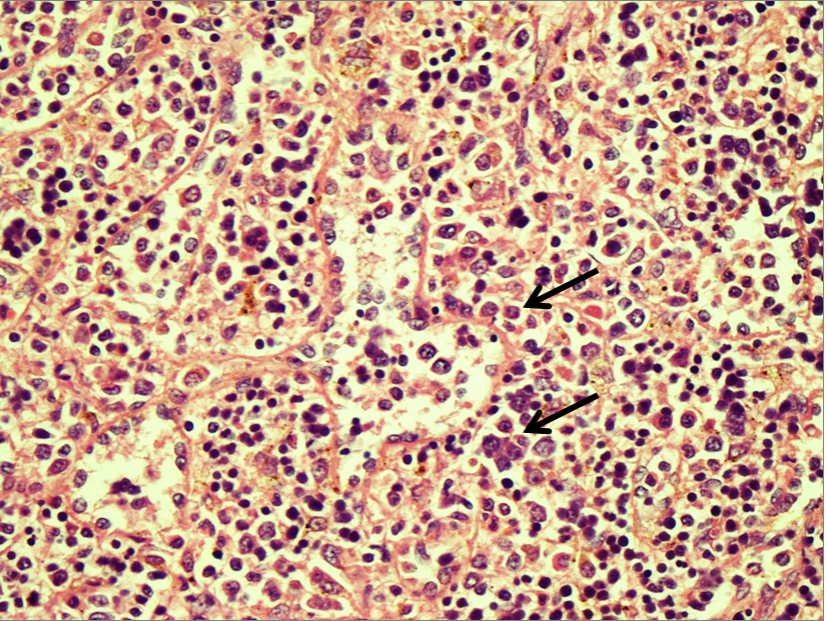
**Specimen of spleen showing numerous erythroid precursors along with occasional megakaryocytes indicating an evidence of hematopoiesis (Arrow)**.

A 20 years old gentleman was being treated for Acute T cell lymphoblastic lymphoma with hyper-CVAD regimen, the therapy consists of Cyclophosphomide, vincristine, doxorubicin and dexamethasone alternating with high dose methotraxate and cytarabine[8]. The patient suffered from severe neutropenia after the first cycle of therapy and required treatment with G-CSF prior to second, with the intention to obtain full benefit of chemotherapy and not compromising on dose. When followed in clinic he showed a low white cell count with an absolute neutrophil count (ANC) of200 cells/microL on day 11 after cycle 2 despite being on G-CSF 300 μg/day for 9 days. He was advised to double the dose of G-CSF to 600 μg/day and instructed to return with a complete blood count in three days. About 10 days later he came to emergency room complaining of severe and sharp left upper quadrant pain radiating to shoulder. His white cell count was > 140 × 10^^9^/l. A CT scan was obtained in the emergency room (FIG 1) which showed splenomegaly with high density fluid noted around the spleen and pelvic region most likely representing hemoperitoneum. There was an irregularity noted posteriorly with in the spleen and the location of high density fluid raised the possibility of splenic rupture. Patient was initially treated with intravenous hydration and pain control, G-CSF was stopped. He under went successful splenectomy two days later, histopathology of which confirmed splenic rupture, splenic engorgement and evidence of extramedullary hemopoiesis (Fig 2). Patient's counts returned to < 14 × 10^9^/l, and he was discharged uneventfully. He is currently completing his treatment with remaining cycles of hyper-CVAD regimen.

## Discussion

Treatment with G-CSF is commonly practiced in patients undergoing neutropenia secondary to chemotherapy for cancers. It is generally considered safe and effective, while many patients have derived benefits of therapy with G-CSF, developing less infection, less antibiotic use and shorter hospital stay, some suffer from minor self limited side effects as well [7]. Prophylactic G-CSF use is recommended when using a chemotherapy regimen associated with a risk of febrile neutropenia in > 20% patients, as is, its use in situations where dose-dense or dose-intense chemotherapy strategies have survival benefit [1]. Uninterrupted G-CSF therapy is recommended in patients undergoing treatment with hyper-CVAD until the white cell count has recovered to 3.0 × 10^9^/L[8]. There have been case reports of splenic rupture in healthy donors of PBSCT patients or patients themselves undergoing mobilization. Two of such patients have reportedly died as a result while the rest were managed successfully with splenectomy [2,9]. Case reports appearing in literature following prophylactic G-CSF use are extremely rare[10].

To the best of our knowledge this is a rare case where G-CSF induced splenic rupture has been reported in patient suffering from acute lymphoblastic lymphoma. The patient presented with acute onset left sided pain without any history of trauma. He uninterruptedly took G-CSF for about 20 days, with a dose of 5 μg/kg for the first ten days followed by 10 μg/kg during the remaining ten. Splenic rupture can be rationalized on the basis of extramedullary myelopoies leading to parenchymal congestion.

Splenomegaly is seen in almost all PBSCT donors receiving G-CSF, regardless of age, sex or race. With a mean size 10.9 ± 2.0 cm before G-CSF to 12.3 ± 2.1 cm on the day of aphaeresis [11]. PBSCT healthy donors receive G-CSF with a dose 10 μg/kg for 5 days only, our patient received on an average a dose of 7.5 μg/kg/day for more than 20 days, which placed him at a high risk for splenic rupture secondary to splenic congestion, despite of the protection offered by the fact that he was neutropenic to begin with (unlike healthy donors). The Rare but serious complication of G-CSF therapy needs to be addressed carefully while treating patients with full intention therapy and a close follow up should be insisted to save the patients form unnecessary hospitalization, cost, morbidity and possibly mortality.

## Consent

"Written informed consent was obtained from the patient for publication of this case report and accompanying images. A copy of the written consent is available for review by the Editor-in-Chief of this journal."

## Competing interests

The authors declare that they have no competing interests.

## Authors' contributions

NM: participated in literature search, manuscript writing and review of document. AJS: participated in manuscript writing, literature search, reviewing the document and coordination with the patient. WAM was the radiologist involved in diagnosis and contributed in literature search. RI was the pathologist involved in diagnosis, reviewing the article and suggesting changes. All authors read the paper and approved the final manuscript.

## About the authors

NM and AJS From the section of oncology, department of medicine: the Aga Khan University hospital, Karachi, Pakistan.

2. WAM from the department of radiology: the Aga Khan University hospital, Karachi, Pakistan.

3. RI from the department of pathology: the Aga Khan University hospital, Karachi, Pakistan.
